# The enigmatic ATP supply of the endoplasmic reticulum

**DOI:** 10.1111/brv.12469

**Published:** 2018-10-19

**Authors:** Maria R. Depaoli, Jesse C. Hay, Wolfgang F. Graier, Roland Malli

**Affiliations:** ^1^ Molecular Biology and Biochemistry, Gottfried Schatz Research Center Medical University of Graz Neue Stiftingtalstraße 6/6, 8010 Graz Austria; ^2^ Division of Biological Sciences and Center for Structural and Functional Neuroscience The University of Montana 32 Campus Drive, HS410, Missoula, MT 59812‐4824 U.S.A.; ^3^ BioTechMed Graz Mozartgasse 12/II, 8010 Graz Austria

**Keywords:** endoplasmic reticulum (ER), ATP, ATP transporter, secretory pathway, protein quality control, ER stress, unfolded protein response (UPR), ERAD

## Abstract

The endoplasmic reticulum (ER) is a functionally and morphologically complex cellular organelle largely responsible for a variety of crucial functions, including protein folding, maturation and degradation. Furthermore, the ER plays an essential role in lipid biosynthesis, dynamic Ca^2+^ storage, and detoxification. Malfunctions in ER‐related processes are responsible for the genesis and progression of many diseases, such as heart failure, cancer, neurodegeneration and metabolic disorders. To fulfill many of its vital functions, the ER relies on a sufficient energy supply in the form of adenosine‐5′‐triphosphate (ATP), the main cellular energy source. Despite landmark discoveries and clarification of the functional principles of ER‐resident proteins and key ER‐related processes, the mechanism underlying ER ATP transport remains somewhat enigmatic. Here we summarize ER‐related ATP‐consuming processes and outline our knowledge about the nature and function of the ER energy supply.

## I. INTRODUCTION

In 1944, a ‘lace‐like reticulum’ appeared on electron micrographs taken by Keith Porter, Albert Claude, and Ernest Fullam, who were the first to describe the network‐resembling structure of the largest membrane‐bound organelle of the eukaryotic cell (Porter, Claude & Fullam, 1945). During the characterization of their discovery, Porter coined the name ‘endoplasmic reticulum’ (Porter & Kallman, 1952), referring to its localization in the central or ‘endoplasmic’ portions of the cytoplasm and its absence from the exterior parts of the ectoplasm. The endoplasmic reticulum (ER) can be found as flat cisternae as well as highly curved tubules comprising a single lumen enclosed by a single membrane (Friedman & Voeltz, 2011). This membrane is continuous with the outer membrane of the nucleus and thus links the two compartments – a connection which becomes evident when considering that a single set of ER proteins participates both in shaping the ER and in building the nuclear pore complex (Dawson *et al*., 2009). In general, contact sites between organelles are important in the coordination of cellular pathways and hence in the maintenance of cellular integrity. Whereas vesicle transport and cell signalling provide the opportunity for long‐distance conversations, the more direct interaction *via* membrane contact sites further promotes this interconnection. A well‐studied and remarkable example for interorganellar contact sites is provided by the so‐called mitochondria‐associated ER membranes (MAMs), which form a distinct subdomain of the smooth ER dedicated to the interaction with mitochondria. MAMs are thereby engaged in a variety of processes ranging from Ca^2+^ signalling and lipid metabolism to mitochondrial fission and inflammasome formation (Raturi & Simmen, 2013; Marchi, Patergnani & Pinton, 2014; Vance, 2014). The ER is divided into two major structural domains called the rough ER and the smooth ER, which can be further subdivided into functional membrane domains such as MAMs, ER quality‐control compartment (ERQC), ER exit sites (ERES) and plasma membrane‐associated membranes (PAMs) (Lynes & Simmen, 2011). The rough ER is defined by its association with ribosomes and is characterized by its involvement in the synthesis of secretory and membrane proteins and major co‐ and post‐translational modifications such as protein folding, glycosylation, secretion and degradation. The smooth ER, on the other hand, is concerned with lipid synthesis, carbohydrate metabolism and Ca^2+^ storage. Considering the diversity of its duties, any malfunction of the ER may have severe or even lethal effects on the cell. Defects in protein secretion or degradation, for example, lead to the accumulation of un‐ or misfolded proteins, causing so‐called ER storage diseases (Rutishauser & Spiess, 2002). Under normal circumstances ER quality control only allows correctly folded proteins to move on to the Golgi, whereas un‐ or misfolded proteins are targeted for degradation by the proteasome. Despite this efficient quality control system, unfavourable conditions can cause the accumulation of too much protein waste, which then triggers a series of signalling pathways collectively known as the unfolded protein response (UPR), leading to the activation of protective cellular mechanisms; when all of these measures fail, prolonged UPR signalling triggers apoptosis and the cells die (Rasheva & Domingos, 2009; Korennykh & Walter, 2012). Thus the ER not only provides a wide set of molecular chaperones and elaborate strategies to control protein folding but also a fallback plan (Bukau, Weissman & Horwich, 2006; Braakman & Bulleid, 2011; Gidalevitz, Stevens & Argon, 2013).

Notably, all of these processes are dependent on an energy supply in the form of ATP, and on the ATP consumed by protein phosphorylation. The first part of this review will describe the contribution of these processes to the energy demand of the ER with an attempt to identify those proteins which actually bind and hydrolyse ATP. The second part will then focus on the fueling of these processes with ATP. Remarkably, both the size and charge of the nucleotide, as well as the ER's inability to generate ATP by itself, make a specific mechanism for its transport into the lumen necessary. Yet little is known about ER ATP import (Hirschberg, Robbins & Abeijon, 1998; Csala *et al*., 2007) with only a few exceptions; these will be discussed critically, taking into consideration the existing knowledge of ER ATP dynamics as well as already identified ATP transporters in other organelles.

## II. ATP‐CONSUMING PROCESSES IN THE ER

**Figure 1:**
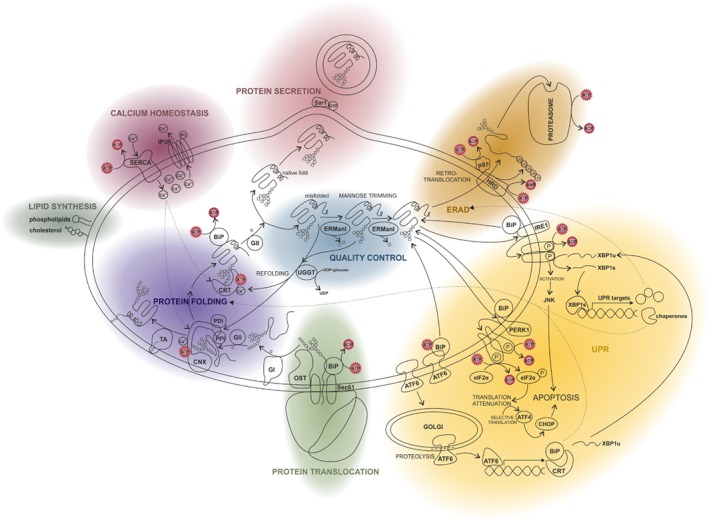
ATP‐consuming processes in the endoplasmic reticulum (ER). Schematic representation providing an overview of the most important ER‐associated processes and showing where ATP is required. Related functions are highlighted in the same colour. In green we follow protein translocation into the ER. Thereafter proteins are modified and folded (lilac). During protein quality control (blue) the folding state of the protein is monitored. Correctly folded proteins are secreted and transported to the Golgi (red). Unfolded and misfolded proteins are refolded. If all folding attempts fail, proteins undergo ER‐associated protein degradation (ERAD, orange). They are ubiquitinated and transferred into the cytosol *via* retrotranslocation, where they are degraded by the proteasome. The accumulation of misfolded proteins in the ER may also trigger the unfolded protein response (UPR, yellow). The UPR includes the activation of the kinases IRE1 and PERK1. IRE1 signalling results in the activation of the transcription factor XBP1 and the subsequent expression of UPR targets such as ER chaperones and ERAD factors. PERK1 causes translation inhibition by eIF2α phosphorylation leading to the successive expression of the transcription factors ATF4 and CHOP, which activates apoptosis‐promoting genes. The third important UPR mediator is the transcription factor ATF6 which induces the transcription of its target genes encoding for instance XBP1, BiP or calreticulin. Beside its central functions in protein synthesis, folding and degradation, the ER is also the most important Ca^2+^ store with an essential contribution to Ca^2+^ signalling events (magenta). Ca^2+^ is pumped into the ER by the ATPase SERCA and is primarily released *via* the IP3 receptor channel. Finally, the ER is mainly responsible for the biosynthesis of phospholipids as well as isoprenoids like cholesterol and steroid hormones (grey). ADP, adenosine diphosphate; ATF4, activating transcription factor 4; ATF6, activating transcription factor 6; ATP, adenosine triphosphate; BiP, binding immunoglobulin protein; CHOP, C/EBP homologous protein; CNX, calnexin; CRT, calreticulin; eIF2α, eukaryotic initiation factor 2α; ERManI, ER mannosidase I; GI, glucosidase I; GII, glucosidase II; GTP, guanosine triphosphate; HRD, ubiquitin‐protein ligase; IP3, inositol trisphosphate; IP3R, inositol triphosphate receptor; IRE1, inositol‐requiring enzyme 1; JNK, c‐Jun N‐terminal kinase; OST, oligosaccharyltransferase; P, indicates phosphorylation; PDI, protein disulfide isomerase; PERK1, proline‐rich receptor‐like protein kinase 1; PPI, prolyl peptidyl isomerase; Sar1, COPII‐associated small GTPase; Sec61, ER membrane protein translocator (translocon); SERCA, sarco/endoplasmic reticulum Ca^2+^‐ATPase; TA, GPI transamidase; UGGT, UDP‐glucose glycoprotein glucosyltransferase; UPR, unfolded protein response; XBP1, x‐box binding protein 1.

The fate of ATP within the ER is now explored by dissecting the functions of the ER (summarized in Fig. 1). Only those processes which take place within the ER lumen require ATP import, but for the sake of completeness ATP‐consuming events on the cytosolic side of the ER membrane are considered as well.

### (1) Protein synthesis and the secretory pathway

Protein synthesis requires energy and the demand for ATP as an energy source is most pronounced at the transcription stage. However, the first ER‐related step of protein synthesis, which is the translation of membrane and secretory proteins by ER‐bound ribosomes, is driven by GTP hydrolysis. Therefore the ER ATP pool is not affected significantly by protein synthesis until the translocation stage.

The process of translocation involves the insertion of secretory and membrane proteins into the ER and has been reviewed previously (Rapoport, 2007; Dudek *et al*., 2015). Estimates suggest that about a third of the eukaryotic proteome takes the secretory pathway (Kanapin *et al*., 2003). To summarize, translocation starts with the binding of the signal recognition particle (SRP) to the hydrophobic N‐terminal signal sequence of the nascent peptide at the ribosome (von Heijne, 1983, 1984, 1986). In general, the SRP complex then directs the translating ribosomes to the translocon, where the peptide is inserted into a channel comprised of the heterotrimeric Sec61 complex. During translocation, the signal peptide complex cleaves the signal peptide, a reaction independent of ATP (Weihofen *et al*., 2002). The translocation is completed by the polypeptide chain binding protein BiP (binding immunoglobulin protein), a luminal ATP binding member of the heat shock protein 70 (HSP70) family of chaperones (Flynn *et al*., 1991; Lyman & Schekman, 1997; Tyedmers *et al*., 2003). Binding and hydrolysis of ATP by BiP is considered to provide the main driving force for protein insertion into the ER (Glick, 1995; Brodsky, 1996; Dudek *et al*., 2015). BiP is involved in both the gating of the channel and the completion of translocation (Dierks *et al*., 1996; Hamman, Hendershot & Johnson, 1998; Haigh & Johnson, 2002; Alder *et al*., 2005; Schäuble *et al*., 2012). Further chaperones like glucose‐regulated protein 94 (Grp94), which belongs to the Hsp90 family, then also assist in protein folding in an ATP‐dependent manner (see also Section II.2). Hsp70 chaperones commonly depend on two types of co‐chaperones, namely J‐domain proteins of the Hsp40 family which stimulate their ATPase activity, and nucleotide exchange factors (NEFs) which allow transition from the ADP‐ to the ATP‐bound state. In the case of BiP, Grp170 [also known as oxygen‐regulated protein 150 (Orp150) or hypoxia up‐regulated protein 1 (HYOI1)] and the BiP‐associated protein, BAP, also known as Sil1 serve as NEFs and thus control its activity *via* its so‐called ATPase cycle (Behnke, Feige & Hendershot, 2015; Bracher & Verghese, 2015). During this cycle, Hsp70 chaperones exist in an ATP‐bound state with a low affinity for their substrate protein and a high‐affinity ADP‐bound state (Mayer & Bukau, 2005). Thus it would seem natural that protein translocation must be somehow coupled to ATP import.

Beyond its role in translocation, BiP and other Hsp70 proteins are also involved in protein folding under normal conditions or during stress when faced with the accumulation of un‐ or misfolded proteins which have to be kept soluble for degradation (Shiber & Ravid, 2014; Clerico *et al*., 2015). Intriguingly, BiP and other protein‐folding factors are the most abundant ER luminal proteins, which is truly indicative of the ER's dedication to this process (Gidalevitz *et al*., 2013).

Protein translocation is also supported by a number of auxiliary components such as the membrane‐integrated translocating chain‐associated membrane (TRAM) protein (Görlich *et al*., 1992; Voigt *et al*., 1996; Hegde *et al*., 1998), the translocon‐associated protein (TRAP) which contains a transmembrane region and a luminal domain located directly below the channel (Ménétret *et al*., 2000, 2008; Fons, Bogert & Hegde, 2003), and the Sec62/Sec63 complex (Meyer *et al*., 2000; Tyedmers *et al*., 2003; Conti *et al*., 2015). The latter functions as a co‐chaperone for BiP by stimulating its ATPase activity *via* the J‐domain of the Hsp40 family member Sec63 (Misselwitz, Staeck & Rapoport, 1998; Rapoport, 2007). Another co‐chaperone of BiP is the Sec63‐related Hsp40 chaperone ER‐resident J‐domain protein 1 (ERj1), which again harbours a J‐domain for the interaction with BiP (Blau *et al*., 2005; Benedix *et al*., 2010). ERj1 can also associate with the ribosome and Sec61 and in this way contributes to translation regulation (Blau *et al*., 2005; Benedix *et al*., 2010). Hence ERj1 is also referred to as ribosome‐associated membrane protein (RAMP), similarly to RAMP4, also known as stress‐associated endoplasmic reticulum protein 1 (SERP1) which participates in translocation as well (Schröder *et al*., 1999; Yamaguchi *et al*., 1999). Other factors related to translocation are protein associated with the ER translocon (PAT‐10) (Meacock *et al*., 2002) and (palmitoylated) calnexin (Lakkaraju *et al*., 2012).

The membrane protein calnexin and its soluble paralogue calreticulin are both Ca^2+^‐dependent ER‐resident chaperones with a lectin site which enables them to bind to glycosylated proteins during and after translocation to prevent their aggregation (Molinari *et al*., 2004; Williams, 2006). Both proteins seem to constitute the core element of a protein‐folding complex. They interact with the ER thiol oxidoreductase ERp57, a protein disulfide isomerase (PDI) which facilitates the formation of disulfide bonds within glycoproteins, as well as with the peptidyl prolyl isomerase (PPI) cyclophilin B. Together with Hsp70 and Hsp90 chaperones, the ER‐located PDIs (Wilkinson & Gilbert, 2004; Galligan & Petersen, 2012) and PPIs (Schiene‐Fischer, 2015) are critical for the folding process. After disulfide formation, the PDI's active centre is reduced and has to be oxidized to restore its activity, which is achieved by the thiol oxidase Ero1. Returning to calnexin and calreticulin, their activity is not only regulated *via* Ca^2+^ binding but also by way of switching between an ATP‐ and an ADP‐bound state. In the case of calreticulin, a very weak ATPase activity of the chaperone itself has been demonstrated, which however might be stimulated by co‐chaperones in a similar fashion to the stimulation of the ATPase activity of Hsp70 proteins by Hsp40 co‐chaperones (Saito *et al*., 1999; Wijeyesakere *et al*., 2015). Calnexin, on the other hand, undergoes conformational changes upon ATP binding, which might also have regulatory consequences (Ou *et al*., 1995). Thus ATP is probably required by both calnexin and calreticulin, albeit their direct or indirect contribution to the overall ATP consumption within the ER is not well characterized.

The recognition of proteins by calnexin and calreticulin depends on their glycosylation. Glycosylation is achieved by the Sec61 neighbouring oligosaccharyltransferase (OST) complex, usually in a co‐translational manner (Kelleher & Gilmore, 2006; Pfeffer *et al*., 2014). The glycosylation state of glycoproteins allows the surveillance of their folding progress. The OST catalyses the transfer of a preassembled glucose_3_‐mannose_9_‐N‐acetylglucosamine_2_ oligosaccharide from dolichol pyrophosphate onto asparagine residues of nascent proteins. During maturation, the oligosaccharide is then trimmed by glucosidase I and II starting with the two terminal glucose residues. The remaining glucose_1_‐mannose_9_‐N‐acetylglucosamine_2_ oligosaccharide is recognized by calnexin or calreticulin which favour proper protein folding. Next, the final glucose residue is also removed. At this point the UDP–glucose glycoprotein glucosyltransferase (UGGT) assumes control. As another protein with chaperone function, UGGT is capable of sensing the folding state of a protein. When they are not correctly folded, UGGT attaches a glucose residue to the N‐terminal glycan. This enables the protein's reassociation with calnexin or calreticulin and thus, ideally, its correct refolding. The repeated association of calnexin or calreticulin and UGGT is described as the calnexin/calreticulin cycle and contributes crucially to glycoprotein folding (Helenius & Aebi, 2004; Caramelo & Parodi, 2008). As soon as proteins reach their native state they exit this cycle and continue to pursue the secretory pathway. However, if the folding process is not successful, proteins are degraded by the ER‐associated protein degradation (ERAD) pathway (see Section II.2*b*).

The oligosaccharide donor dolichol pyrophosphate provides a lipid anchor which allows the proper positioning of the oligosaccharide at the ER membrane next to the OST complex. The biosynthesis of dolichol comes within the mevalonate pathway where isopentenyl diphosphate (IPP) molecules are produced (Ferguson, Durr & Rudney, 1959). An ER‐resident isoprenyltransferase then catalyses a condensation reaction between varying numbers of IPPs with farnesyl diphosphate (FPP) to build polyprenyl diphosphate, which is further converted to dolichol in several reaction steps (Crick, Rush & Waechter, 1991; Schenk, Fernandez & Waechter, 2001a; Schenk *et al*., 2001b). The oligosaccharide is assembled by the sequential addition of monosaccharides (mannose, N‐acetylglucosamine or glucose) to dolichol pyrophosphate by glycosyltransferases, which takes place on the cytosolic and the luminal side of the ER membrane (Kelleher & Gilmore, 2006). While ATP is used during the mevalonate pathway, it does not seem to be required for the activity of the OST in the ER.

About 10–20% of all membrane proteins that take the secretory pathway are additionally modified within the ER by the C‐terminal attachment of a glycosylphosphatidylinositol (GPI) anchor (Orlean & Menon, 2007; Kinoshita & Fujita, 2016). In an analogous mode to the dolichol oligosaccharide, the GPI anchor is preassembled by ER‐membrane and luminal enzymes. The assembly pathway is initialized at the cytosolic face of the ER and is completed in the lumen after the seemingly ATP‐independent flipping of the anchor to the other side (Vishwakarma & Menon, 2005). After completion, the GPI anchor is finally transferred to proteins with the respective target sequence by the GPI transamidase complex.

Once the ER‐located folding machinery has successfully completed its work, proteins are packed into coat protein complex II (COPII)‐coated vesicles which are transported to the Golgi (D'Arcangelo, Stahmer & Miller, 2013; Saito & Katada, 2015). The formation of vesicles starts with the assembly of coat proteins on the cytosolic side of the ER membrane, which causes membrane curvature and formation of a vesicle bud (Barlowe *et al*., 1994). This process and the subsequent packing of the cargo are directed by the GTPase secretion‐associated RAS‐related protein 1(Sar1) in cooperation with the guanosine exchange factor (GEF) Sec12 and the GTPase‐activating protein (GAP) Sec23 (Barlowe & Schekman, 1993; Antonny *et al*., 2001). GTP binding by Sar1 initializes coat formation while GTP hydrolysis is required for effective cargo sorting and vesicle fission (Bielli *et al*., 2005). The cargo proteins either passively diffuse into the forming vesicle bud or are actively recruited by the adaptor protein Sec24 to be incorporated into the nascent vesicle (Miller *et al*., 2002, 2003).

Vesicle formation takes place at specific sites of the ER which are termed transitional ER or ERESs and can be defined by the presence of the Sec16 protein (Watson *et al*., 2006; Hughes *et al*., 2009). Targeting to and fusion with the Golgi is mediated by Rab (Ras‐related in brain) proteins and soluble N‐ethylmaleimide‐sensitive‐factor attachment receptor (SNARE) complex formation (Allan, Moyer & Balch, 2000; Moyer, Allan & Balch, 2001; Ortiz Sandoval & Simmen, 2012). The secretory pathway will not be discussed in more detail here as our focus is the ER, but more information can be found, for example, in a comprehensive review by Barlowe & Miller (2013).

### (2) Protein quality control and ER stress

The accumulation of un‐ or misfolded proteins as a result of disturbing events like mutant protein expression, nutrient deprivation, viral infection or intoxication commonly causes ER stress, which is sensed by a specialized ER‐associated machinery. Misfolded proteins may be refolded or, if that fails, exported into the cytosol by a mechanism called retrotranslocation, followed by their degradation by the proteasome. This process, referred to as ERAD, together with the UPR, are considered to be the most important mechanisms of protein quality control activated upon ER stress (Bukau *et al*., 2006).

#### (a) *The unfolded protein response (UPR)*

Several signalling pathways are responsible for the surveillance of the ER folding machinery. The components of these pathways sense the accumulation of un‐ or misfolded proteins and induce a series of different response mechanisms which increase the ER folding capacity and are collectively described as the UPR (Schröder & Kaufman, 2005; Rasheva & Domingos, 2009; Korennykh & Walter, 2012). In mammals, the UPR is initiated by at least three ER transmembrane proteins, the kinases inositol‐requiring enzyme 1 (IRE1) (Chen & Brandizzi, 2013) and proline‐rich receptor‐like protein kinase 1 (PERK1) as well as the transcription factor activating transcription factor 6 (ATF6).

IRE1 is able to sense ER folding capacity *via* its luminal domain and transmits the signal to its cytosolic kinase and ribonuclease domains. ER stress induces the oligomerization of IRE1 proteins, leading to activation of the IRE1 kinase. As a consequence, the ribonuclease domain is activated and cleaves the messenger RNA (mRNA) of a basic leucine zipper (b‐Zip) transcription factor [Hac1 in yeast, X‐box binding protein 1(XBP1) in metazoans] (Yoshida *et al*., 2001). The cleavage allows the translation of the active form of XBP1/Hac1, which then induces the expression of UPR targets such as ER chaperones and ERAD factors. The second UPR‐connected kinase is PERK1. PERK1 harbours a luminal domain highly similar to that of IRE1, suggesting a similar sensing mechanism. However, it is not known with certainty how IRE1 and PERK1 monitor ER stress. There is evidence that BiP inactivates them by binding to their luminal domain; accumulated un‐ or misfolded proteins may then titrate BiP from this binding site, leading to the activation of IRE1 and PERK1 (Oikawa *et al*., 2009, 2012; Pincus *et al*., 2010). Although the role of BiP is not understood completely, two factors that could influence its dissociation from IRE1 and PERK1 are the availability of unfolded protein targets, and manipulation of its ATPase cycle by nucleotide levels or cofactors (Amin‐Wetzel *et al*., 2017). On the other hand, it is also possible that stress‐sensor activation occurs directly upon binding of un‐ or misfolded proteins to their luminal domain, which indeed contains a hydrophobic peptide binding domain (Credle *et al*., 2005; Gardner & Walter, 2011). Unlike that of IRE1, the cytosolic domain of PERK1 contains a eukaryotic initiation factor 2α (eIF2α) kinase, implicating a mechanism for translation regulation (Shi *et al*., 1998, 1999; Harding, Zhang & Ron, 1999; Harding *et al*., 2000; Marciniak *et al*., 2006): i.e. phosphorylation of eIF2α leads to its inactivation and hence inhibits translation initiation. The inhibition of translation makes sense as a consequence of accumulated misfolded proteins since the production of new proteins would only contribute to the aggravation of the situation by occupying the ER folding machinery. The third important mediator of the UPR, ATF6, is delivered to the Golgi upon ER stress (Haze *et al*., 1999; Wang *et al*., 2000). For that to happen, BiP has to dissociate from ATF6 to uncover its Golgi localization signal (Shen *et al*., 2002, 2005). The mechanism behind BiP dissociation from ATF6 is not clear, but one possibility is that ER stress actively dissociates BiP from ATF6 by activating the BiP ATPase cycle (Shen *et al*., 2005). In the Golgi, ATF6 is cleaved by resident proteases, which results in the release of the transcription factor domain from the transmembrane domain. The transcription factor domain then enters the nucleus and activates its target genes, encoding for instance XBP1, BiP or calreticulin. Since BiP activity is controlled by its ATPase cycle, its involvement in the sensing mechanism of all three UPR mediators entails the need for ATP during the initiating steps of the UPR. However, the signalling events themselves are not likely to consume notable amounts of ATP within the ER, even though IRE1 and PERK1 require ATP at the cytosolic face of the ER membrane for their kinase activity. In addition, UPR signalling ultimately results in increased production of ATP‐consuming chaperones, while translation, in general, is reduced, suggesting a reduced level of ATP hydrolysis for that purpose. Considering this, the ATP consumption rate might vary according to the phase of the UPR.

#### (b) *ER‐associated protein degradation (ERAD)*

Besides the UPR signalling events, which contribute to the restoration of regular protein folding, the removal of accumulated protein waste itself is accomplished by another set of processes involved in ER protein control, ERAD (Meusser *et al*., 2005; Hirsch *et al*., 2009). Briefly, ERAD starts with the recognition of un‐ or misfolded proteins, mainly by chaperones of the Hsp70 family. They are then targeted to membrane‐embedded ubiquitin ligases (Carvalho, Goder & Rapoport, 2006) such as the yeast ERAD‐associated E3 ubiquitin‐protein ligase (Hrd1) also known as degradation in the endoplasmic reticulum protein 1 (Der1) ligase (Bays *et al*., 2001; Deak & Wolf, 2001; Mehnert, Sommer & Jarosch, 2014) or its human homologues HRD1 (Kaneko *et al*., 2002; Nadav *et al*., 2003) and glycoprotein 78 (gp78) (Fang *et al*., 2001). After their dislocation to the cytosolic side of the ER membrane, proteins are ubiquitinated by ubiquitin ligase complexes, a reaction which requires energy in the form of ATP at the ER membrane. Ubiquitinated proteins are then released into the cytosol, which again requires ATP hydrolysis at the ER membrane for the activity of the cytosolic ATPase associated with diverse cellular activities (AAA‐ATPase) p97, referred to as cell division protein 48 (Cdc48) in yeast. The latter converts the energy released by ATP hydrolysis into the mechanical force needed for pulling proteins out of the membrane (Ye, Meyer & Rapoport, 2001). In the cytosol, proteins are degraded by the proteasome, which requires ATP hydrolysis as well. Here, ATP hydrolysis is not actually spatially linked to the ER but closely coupled with an ER process in a temporal manner (Amm, Sommer & Wolf, 2014).

An essential step during ERAD is the export of proteins into the cytosol, which requires the crossing of the ER membrane and is referred to as retrotranslocation or dislocation. To date, the identity of a retrotranslocation channel has not been determined with certainty (Hampton & Sommer, 2012). It has been suggested that the Sec61 translocation channel is directly involved in both retrotranslocation as well as translocation (Wiertz *et al*., 1996; Pilon, Schekman & Römisch, 1997; Plemper *et al*., 1997; Zhou & Schekman, 1999). On the other hand, the HRD ligase complex, which is already responsible for substrate recognition and ubiquitination, may also provide the conduit for retrotranslocation. In this context, there is also evidence to suggest that the HRD‐associated complex composed of Derlin‐1, the ATPase p97, and VCP‐interacting membrane protein (VIMP) mediates retrotranslocation (Lilley & Ploegh, 2004; Ye *et al*., 2004; Lilley & Ploegh, 2005). Furthermore, an alternative model was proposed by Ploegh (2007) suggesting that lipid droplet formation was involved in the removal of misfolded proteins from the ER.

The recognition of misfolded proteins relies on the N‐linked glycan which is attached to most secretory proteins in the course of translocation (Moremen & Molinari, 2006; Lederkremer, 2009; Määttänen *et al*., 2010; Yoshida & Tanaka, 2010). Within the ER all three glucose residues of the oligosaccharide are removed unless the protein cannot be successfully folded. Proteins that are not successfully folded repeatedly associate with the lectin‐like chaperones calnexin or calreticulin to allow refolding. However, if the folding process takes too long, a timer mechanism involving the ER mannosidase I (ERManI) and mannosidase like proteins (EDEMs) initiates ERAD (Hosokawa *et al*., 2001; Herscovics, Romero & Tremblay, 2002; Molinari *et al*., 2003; Oda *et al*., 2003; Olivari & Molinari, 2007). After a certain residence time in the ER, ERManI removes the terminal mannose residue of all glycosylated proteins, which serves as the mechanism for escaping further folding attempts (Frenkel *et al*., 2003). With no mannose left, UGGT can no longer add glucose to the oligosaccharide, which obstructs the association of misfolded proteins with calnexin or calreticulin and probably targets them for degradation. Hence proteins will be degraded if they are not properly folded within a certain time frame. Notably, ERAD processes appear to be preferentially localized to certain regions within the ER – the so‐called ER‐derived quality control compartment (ERQC) (Kamhi‐Nesher *et al*., 2001). In this pericentriolar compartment ERAD substrates as well as calnexin and calreticulin, PDIs, UGGT, and ERManI, but not BiP are accumulated (Frenkel *et al*., 2004; Avezov *et al*., 2008; Benyair *et al*., 2015). This compartmentalization may also contribute to the regulation of ERAD. Another recognition mechanism engages other ER‐resident as well as cytosolic chaperones (Nishikawa, Brodsky & Nakatsukasa, 2005; Brodsky, 2007). Among the most prominent examples is BiP, which seems to participate in all stages of protein maturation and degradation (Otero, Lizák & Hendershot, 2010), supported by other Hsp70 chaperones as well as Hsp90 chaperones such as Grp94 (Christianson *et al*., 2008, 2012; Eletto, Dersh & Argon, 2010) and PDIs (Feige & Hendershot, 2011; Suzuki & Schmitt, 2015).

#### (c) *ER stress‐induced apoptosis*

If all attempts to eliminate ER stress fail, the continued accumulation of misfolded proteins induces cell death by apoptosis (Rasheva & Domingos, 2009). The exact mechanism by which ER stress regulates apoptosis is not well known, but it seems that apoptosis follows sustained PERK1 but not IRE1 signalling (Lin *et al*., 2009). PERK1‐induced phosphorylation of eIF2α prevents the synthesis of most proteins. However, translation of the transcription factor ATF4, immune to this inhibition, is selectively increased (Harding *et al*., 2000; Lu, Harding & Ron, 2004; Vattem & Wek, 2004). ATF4 now induces the expression of C/EBP homologous protein (CHOP), another transcription factor, which evidently promotes apoptosis in response to ER malfunction (Zinszner *et al*., 1998; Fawcett *et al*., 1999). Apoptosis induction is achieved at least in part by the CHOP‐mediated upregulation of the pro‐apoptotic factor Bim and the downregulation of the anti‐apoptotic B‐cell lymphoma 2 (Bcl2) (McCullough *et al*., 2001; Puthalakath *et al*., 2007). Furthermore, the expression of the ER thiol oxidase Ero1 (see also Section II.1) is increased; Ero1 normally promotes protein folding by disulfide formation, but also has pro‐apoptotic functions probably by producing reactive oxygen species (ROS) during this reaction (Marciniak *et al*., 2004). However, IRE1 signalling may also contribute to apoptosis induction *via* activation of the c‐Jun N‐terminal kinases (JNK) pathway (Urano *et al*., 2000). And finally, Ca^2+^ release from the ER and its transfer into mitochondria are considered to mediate apoptosis induction in response to ER stress. This process involves, for instance, the ER Ca^2+^‐ATPase S1T, a truncated version of the ER Ca^2+^ pump sarco/endoplasmic reticulum Ca^2+^‐ATPase (SERCA1) (see also Section II.3), which is induced *via* the PERK1‐eIF2α‐ATF4 pathway (Chami *et al*., 2008). The activity of S1T, which is localized at ER–mitochondria contact sites, leads to ER Ca^2+^ depletion and increases mitochondria contact sites with the overall result of increased Ca^2+^ transfer from the ER to mitochondria. The strong accumulation of Ca^2+^ in the mitochondria subsequently induces apoptosis *via* the opening of the mitochondrial permeability transition pore (PTP) (Pizzo & Pozzan, 2007; Pinton *et al*., 2008).

### (3) Ca^2+^ homeostasis

The role of the ER as the main Ca^2+^ store determines its importance for all processes dependent on Ca^2+^ signalling, like protein folding by the Ca^2+^‐dependent chaperones calreticulin and calnexin or the induction of UPR and apoptosis as described above and reviewed by Krebs, Agellon & Michalak (2015). The release of Ca^2+^ from the stores, on the other hand, is mediated by inositol 1,4,5‐trisphosphate (InsP_3_) or ryanodine receptor channels. A potential regulatory connection of cellular ATP levels to ER Ca^2+^ homeostasis is that the InsP_3_ receptor channel‐opening probability is increased by high cytosolic ATP concentrations (Betzenhauser *et al*., 2008). The rapid mobilization of Ca^2+^ by InsP_3_ and ryanodine receptors is followed by a phase of slow entry of extracellular Ca^2+^ (Clapham, 2007). Depleted ER stores then have to be replenished, which is accomplished by a renewed entry of extracellular Ca^2+^. The second Ca^2+^ entry phase furthermore provides a more long‐lasting signal. Refilling of the ER is achieved by the SERCA, a P‐type ATPase, which uses ATP to pump Ca^2+^ against the ion concentration gradient. It should be pointed out here that owing to the role of Ca^2+^ as a regulator of ER‐associated processes, changing Ca^2+^ levels within the ER may somehow correlate with ATP concentration and transport.

### (4) Lipid biosynthesis

The ER can be considered a highly productive compartment as it is crucially involved in the supply of the cell with various molecular components such as proteins, as described above. In addition, lipid synthesis also takes place largely in the (smooth) ER, including the synthesis of phospholipids (Vance, 2015) and isoprenoids such as cholesterol and steroid hormones (Holstein & Hohl, 2004).

During phospholipid synthesis, first phosphatidic acid is produced from glycerol‐3‐phosphate or dihydroxyacetone‐phosphate by the action of acyltransferases (Vance, 2015). Phosphatidic acid is then converted to diacylglycerol by phosphatidic acid phosphatase‐1 or reacts with CTP to form CDP‐diacylglycerol catalysed by CDP‐diacylglycerol synthase. The energy for the transfer reactions is provided by the use of acyl‐coenzyme A (acyl‐CoA) as the fatty acid donor. Another step in phospholipid synthesis is the formation of the headgroups and their transfer onto phosphatidic acid. Phosphatidylcholine (PC), for example, is formed *via* the so‐called Kennedy pathway (Kennedy & Weiss, 1956). After choline has been imported into the cell it is phosphorylated by the cytosolic choline kinase followed by the generation of CDP‐choline by the CTP:phosphocholine cytidylyltransferase which is located in the nucleus and cytosol. Finally, an ER membrane transferase catalyses the transfer of CDP‐choline to diacylglycerol to result in PC. The synthesis of phosphatidylethanolamine (PE) is analogous to PC synthesis starting with the phosphorylation of ethanolamine by a cytosolic kinase followed by the conversion into CDP‐ethanolamine by the CTP:phosphoethanolamine cytidylyltransferase and the transfer onto diacylglycerol. The second pathway for PE synthesis includes the mitochondrial phosphatidylserine (PS) decarboxylase which decarboxylates PS to PE (Borkenhagen, Kennedy & Fielding, 1961). PC and PE can now be converted into PS by replacing the choline (PS synthase‐1) or ethanolamine (PS synthase‐2) with serine. Both PS synthases are enriched in MAMs (Stone & Vance, 2000). In similar fashion the production of phosphatidylinositol (PI) and phosphoglycerol and its derivatives cardiolipin and lyso(bis)phosphatidic acid are mainly attributed to ER and secondarily to mitochondrial membranes. In summary, although the enzymes required for the production of phospholipids are primarily found within the ER membrane or activated there, the ER ATP pool is probably not affected significantly by phospholipid synthesis. On the other hand, the activation of fatty acids to acyl‐CoA as well as the phosphorylation of choline and ethanolamine require ATP outside of the lumen.

Since the ER is the main location of phospholipid synthesis, their transport to other membranes is another task for the cell. The mechanism of phospholipid transfer remains rather enigmatic. Vance (2015) discusses three possible mechanisms: transport through the cytosol *via* carrier proteins, transport in phospholipid vesicles, or direct exchange *via* membrane contact sites such as MAMs. However, since very little is known regarding this mechanism, the impact on energy consumption likewise remains obscure.

Isoprenoid synthesis first requires the formation of IPP by the mevalonate pathway (Ferguson *et al*., 1959), which, as already mentioned, takes place in the cytosol (see also Section II.1). Three molecules of IPP condense to form farnesyl diphosphate, which then reacts with a second molecule of farnesyl diphosphate to form squalene which is inserted into the ER membrane. This reaction is catalysed by farnesyl‐diphosphate farnesyltransferase (squalene synthase), an enzyme located in the ER membrane. The two‐step conversion of squalene to lanosterol and all further 19 reactions required to form cholesterol are also driven by ER membrane enzymes (Gaylor, 2002). Notably, cholesterol biosynthesis as well as the conversion of cholesterol into steroid hormones, which also mostly takes place in the ER, does not require ATP within the ER but rather coenzymes such as nicotinamide adenine dinucleotide (NAD), nicotinamide adenine dinucleotide phosphate (NADP) and flavin adenine dinucleotide (FAD) since most reactions fall into the category of redox reactions.

### (5) ATP‐consuming ER transporters

The ER as an enclosed compartment has to provide entry and exit opportunities for molecules which cannot diffuse through the lipid bilayer. The transport of proteins into and out of the ER in the course of translocation and retrotranslocation was described in Section II.1. However, the wide range of ER‐resident enzymes necessitates the import of substrates and cofactors, such as ATP or NAD(P)(H), into the ER and the export of their products out of the ER. Thus transporters are essential for the maintenance of ER‐associated processes. An overview of ER transporters is given by Csala *et al*. (2007). Here we consider only ATP‐consuming transporters, while the ATP transporter itself will be discussed in Section III.1.

The presentation of peptide antigens by major histocompatibility complex class I (MHC‐I) molecules on the cell surface is a vital mechanism of the immune system (Oliveira & van Hall, 2015). The antigen‐processing pathway starts with the cleavage of proteins by the proteasome. Peptide fragments are then imported into the ER *via* the transporter associated with antigen processing (TAP). In the ER they are trimmed by ER aminopeptidases 1 and 2 (ERAP1/2) and then loaded onto MHC‐I molecules by the peptide‐loading complex (PLC) (Koch & Tampé, 2006). This process is assisted by chaperones including BiP, calnexin, calreticulin, the transmembrane protein tapasin as well as the oxidoreductase ERp57 (see also Section II.1). Finally, the MHC‐I antigen complex is exported from the ER and transported by vesicle transport *via* the trans‐Golgi to the cell surface, where the antigen is presented. The heterodimeric TAP transporter is a member of the ATP‐binding cassette (ABC) family (Ritz & Seliger, 2001; Abele & Tampé, 2011). Binding of a peptide induces ATP hydrolysis at the cytosolic ATP‐binding domains, which drives its import into the ER. Furthermore, luminal ATP is required by chaperones involved in MHC‐I loading, and hence also concerned with the regulation of this process (Wijeyesakere *et al*., 2015).

## III. THE ELUSIVE ER ATP TRANSPORTER

All of the above‐described essential ER‐dependent cellular processes determine the energy demand of the ER and the requirement for ATP import. The magnitude of this demand, however, is subject to variations in the dynamics of these processes. Thus ATP import into the ER not only requires a means of transport but will also be subject to regulatory events. However, in mammalian cells to date, neither has the ATP transport mechanism been identified nor have ATP dynamics within the ER been accurately described. We now attempt to summarize past endeavours to identify an ER ATP transporter as well as providing possible avenues for future studies.

### (1) A presumed ER ATP transport protein – characterization of ATP transport into rat liver ER

**Figure 2:**
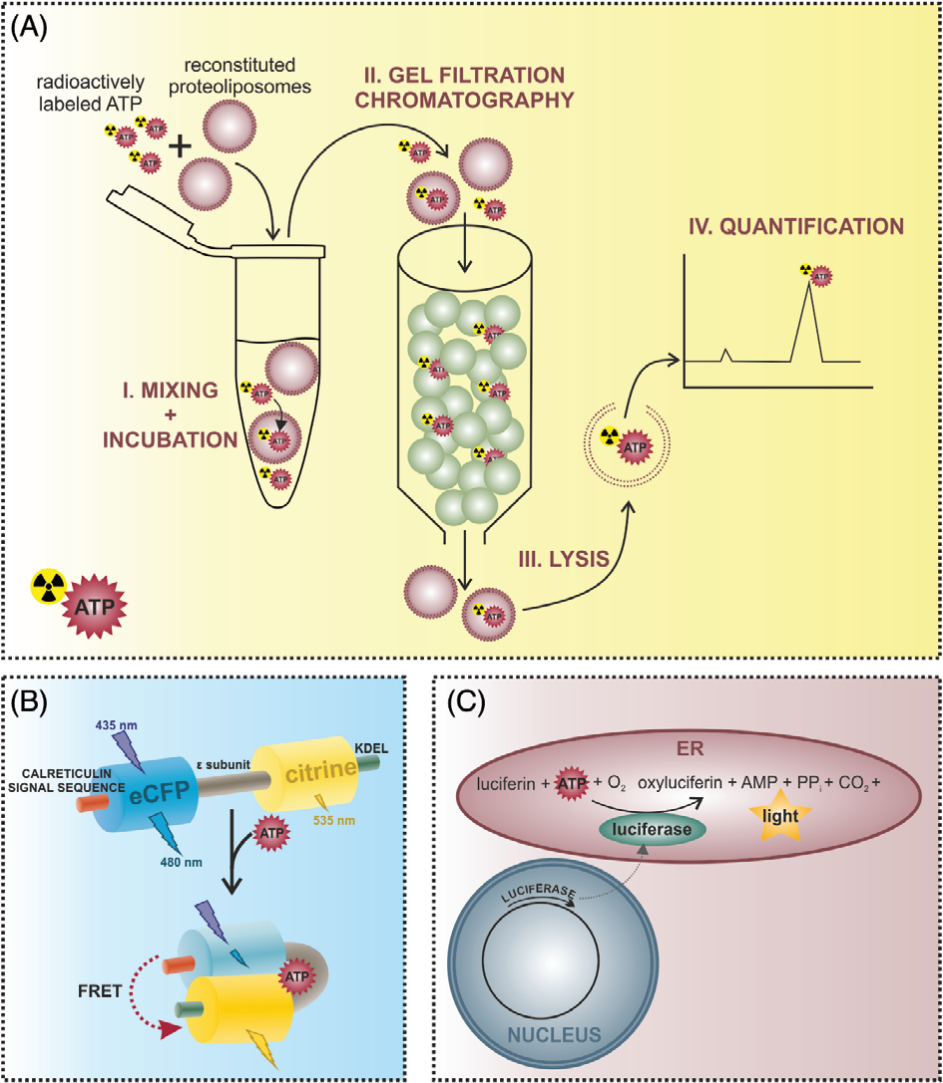
Methods for the investigation of endoplasmic reticulum (ER) ATP dynamics. (A) ATP transport into ER‐derived vesicles or reconstituted liposomes containing ER proteins can be measured using an *in vitro* approach. Proteoliposomes are incubated with radioactively labeled ATP and then applied to a gel filtration column. Free ATP binds to the matrix, while proteoliposomes and incorporated ATP are eluted in the void volume. Proteoliposomes are then lysed to determine the amount of imported ATP *via* high‐performance liquid chromatography (HPLC) or liquid scintillation counting. (B) Alterations of ER ATP levels can be measured using genetically encoded fluorescent ATP sensors targeted to the ER. The ERAT4.01 probe for example is a Förster/fluorescence resonance energy transfer (FRET)‐based sensor consisting of two fluorescent proteins – an enhanced cyan fluorescent protein (CFP) and the yellow fluorescent protein (YFP) variant citrine – flanking the ATP binding ϵ‐subunit of the F_o_F_1_‐ATP synthase from *Bacillus subtilis* (Imamura *et al*., 2009; Vishnu *et al*., 2014). The N‐terminal fusion of the calreticulin signal sequence and the C‐terminal addition of the ER retention signal with the amino acid sequence KDEL (lysine–aspartic acid–glutamic acid–leucine) allow the efficient targeting of the probe to the ER. Binding of ATP to the ϵ‐subunit causes a conformational change of the sensor, which increases the FRET signal intensity. (C) Alternatively, ER ATP levels can be determined using an ER‐targeted heterologously expressed firefly luciferase, which catalyses the reaction of oxyluciferin to luciferin, resulting in light emission. Since this reaction is ATP dependent, the intensity of the emitted light is proportional to the ATP concentration.

In the early 1990s, promising results regarding the transport of ATP into the ER formed the foundation for the elucidation of the underlying mechanism. Clairmont, Maio & Hirschberg (1992) demonstrated the translocation of ATP into rough ER‐derived vesicles isolated from rat liver. Because of the many ATP‐binding and ATP‐consuming proteins present in the vesicle membranes, a system based on reconstituted ER proteoliposomes was established to minimize the consumption of ATP during transport assays (see also Section III.7 and Fig. 2A). For this reason, proteins from rat liver ER membranes were reconstituted into phosphatidylcholine liposomes, which were then employed to study the import of ATP into their lumen (Guillén & Hirschberg, 1995). Transport was highly specific, temperature dependent and saturable and could be prevented by the addition of 4,4′‐diisothiocyano‐2,2′‐stilbenedisulfonic acid (DIDS), a known inhibitor of anion exchangers, but not by atractyloside, which inhibits the mitochondrial ATP/ADP translocase (ANT).

Similarly to Clairmont *et al*. (1992), vesicles derived from rat liver rough ER were used by Kim, Shin & Park (1996) in an attempt to identify the presumed ATP transporter using photoaffinity labelling. For this purpose, a photoreactive azido derivative of ATP, 3'‐O‐(p‐azidobenzoyl)‐ATP (AB‐ATP), was synthesized. Rough ER vesicles then were incubated with [γ‐^32^P]AB‐ATP under varying conditions. UV radiation was applied to photolyse [γ‐^32^P]AB‐ATP, thereby converting it into a highly reactive form which permanently binds to and labels associated proteins. For the ER‐derived vesicles, only one protein with a molecular weight of 56 kDa was labelled, and this was declared an ATP transporter candidate. The protein was protected from photoaffinity labelling by adding non‐radioactive ATP but not GTP, demonstrating the specificity of the label for ATP binding proteins. As also reported by Guillén & Hirschberg (1995) for reconstituted ER proteoliposomes, the labelling efficiency was reduced by DIDS, but not affected by atractyloside (Kim *et al*., 1996), suggesting that the same possible transporter was observed in both cases. In further experiments, solubilized rough ER proteins were fractionated using DEAE chromatography and each fraction was photoaffinity labelled and reconstituted in liposomes to test transport activity. As expected the active fraction contained the photolabeled 56 kDa protein (Kim *et al*., 1996).

Finally, Shin *et al*. (2000) reported the characterization of a rat liver ER ATP transporter in reconstituted proteoliposomes. They found that ATP transport was strongly *cis*‐inhibited by ADP and also weakly by AMP and that preincubation of the proteoliposomes with ADP increased the ATP transport activity. Altogether their results suggest an ATP/ADP antiport mechanism in the ER ATP transporter.

### (2) Is membrane trafficking involved in ATP transport? The phosphoinositide phosphatase Sac1 is connected with ER ATP import in yeast

Mayinger & Meyer (1993) described an ATP transport system of vesicles from yeast ER, which was found to be saturable and could be inhibited by DIDS and N‐ethylmaleimide. For the characterization of this system, ER membrane proteins were reconstituted into proteoliposomes to measure ATP transport (see also Section III.7 and Fig. 2A) revealing that ATP uptake was dependent on a counter substrate (ATP or ADP) in the lumen of the vesicle (Mayinger, Bankaitis & Meyer, 1995). It was therefore suggested that ATP import *in vivo* was linked to ADP export, implying the existence of an antiport mechanism. Protein extracts of the proteoliposome membrane were then purified with hydroxyapatite, commonly used for the purification of membrane carrier proteins. The purified fractions were again reconstituted into proteoliposomes to measure ATP transport. The fractions capable of ATP transport were repeatedly subfractionated by chromatography on hydroxyapatite and affinity chromatography with ATP agarose, resulting in a distinct enrichment of the specific ATP transport activity. Sodium dodecyl sulfate–polyacrylamide gel electrophoresis (SDS‐PAGE) analysis of the ultimate fraction allowed the identification of a single protein, namely the phosphatidylinositide phosphatase Sac1, which was thus considered to be involved in ATP transport. It was already known that the *SAC1* gene was genetically linked with *ACT1* (Novick, Osmond & Botstein, 1989) and *SEC14* (Cleves, Novick & Bankaitis, 1989) suggesting its involvement in cytoskeletal organization and the secretory pathway, respectively. The transmembrane protein Sac1 localizes to the Golgi and ER and contributes to the Golgi's secretory function. Its connection with Sec14, which is a phospholipid transfer protein (Bankaitis *et al*., 1990), and the discovery of a phosphatidylinositol phosphate (PtdInsP) phosphatase domain within Sac1, homologous to the mammalian inositol‐5‐phosphatase synaptojanin (McPherson *et al*., 1996), allowed more exact placement of Sac1 in phospholipid metabolism. This domain is conserved from yeast to human and was designated the *SAC1*‐like domain (Guo *et al*., 1999). The functional association of a phosphoinositide phosphatase with the ER and Golgi complex may help us to understand the observed characteristics of Sac1 described above. Phosphoinositides are key players in signal transduction at the plasma membrane as well as the regulation of the actin cytoskeleton, membrane trafficking, and transport. Their precursor form phosphatidylinositol is synthesized in the ER and transferred into other organelles *via* transfer proteins or vesicular transport (Di Paolo & De Camilli, 2006). There are seven different phosphoinositides which are distributed in a characteristic way in a subset of membranes, e.g. phosphatidylinositol 4‐phosphate [PI(4)P] is enriched in the Golgi complex whereas the inositol ring is mostly not phosphorylated in the ER (Balla, 2013). The localization of phosphoinositides correlates with their phosphorylation state which creates a platform for regulatory events through alterations by phosphatases or kinases. Deletions of *SAC1* lead to a massive accumulation of PI(4)P because it cannot be dephosphorylated (Guo *et al*., 1999). This could explain how the phosphatase activity of Sac1 may be involved in the coordination of membrane trafficking between the Golgi and ER, where the dephosphorylation of PI(4)P may occur during retrograde transport from the Golgi to the ER (Di Paolo & De Camilli, 2006; Mayinger, 2012). It might even be possible that such events play a part in ER ATP transport. Further details concerning the role of Sac1 in the ER were revealed by analysis of its contribution to ER ATP transport (Kochendörfer *et al*., 1999). By measuring the transport capacity of isolated microsomal membranes from a *Δsac1* strain compared to wild‐type cells it was shown that Sac1 is required for ER ATP transport. When purified Sac1 protein was added to reconstituted proteoliposomes from *Δsac1* cells, their ability to transport ATP was reestablished. Furthermore, overexpression of Sac1 caused increased ATP uptake. All these observations reinforced the impression that Sac1 contributes in an essential way to the transport of ATP into the ER. However, proteoliposomes containing only Sac1 were not capable of ATP transport, which means that Sac1 itself is not an ATP transporter but rather a cofactor or regulator. In further experiments Kochendörfer *et al*. (1999) demonstrated that *Δsac1* cells exhibit an ER folding defect and constitutive activation of the UPR. It is very likely that these effects are due to reduced luminal ATP levels in *Δsac1* cells, interfering dramatically with the functionality of ATP‐dependent chaperones. Sac1 has also been found to play a significant role in non‐vesicular lipid exchange between the ER and Golgi that may take place at ER/Golgi contact sites. In this case, Sac1 is speculated to help maintain a steep PI(4)P gradient from the Golgi to the ER by dephosphorylating PI(4)P in the ER. This gradient may then be used in a counter‐transport mechanism to move sterols against their gradient from the ER to the Golgi (Del Bel & Brill, 2018). However, how Sac1's roles in vesicle transport and non‐vesicular transport might be mechanistically related to ATP import into the ER remains an unsolved mystery.

Intriguingly, another yeast protein, morphogenesis checkpoint‐dependent protein 4 (Mcd4), has been connected to ATP transport and membrane trafficking (Zhong, Malhotra & Guidotti, 2003). Mcd4, which is found in intracellular membranes, is known to be required for ethanolamine phosphate transfer into the ER during GPI anchor synthesis (see Section II.1). However, Mcd4 is also able to trigger extracellular ATP release in a process mediated *via* the membrane trafficking pathway (Zhong *et al*., 2003). This process also involves the transport of ATP into the Golgi and the contribution of the vacuolar H^+^‐ATPase. As in the case of the Sac1 protein described above, these observations support a speculated connection between membrane trafficking and ATP transport. Moreover, the ER might also serve as an intermediate storage compartment for ATP for release into the extracellular space. A possible ATP transporter for vesicles has been identified in the form of solute carrier family 17 member 9 (SLC17A9) (Sawada *et al*., 2008). It is possible that this transporter also takes part in the transport of ATP *via* vesicles.

### (3) An actual ER ATP transporter – the discovery of the *Arabidopsis thaliana* ER ATP/ADP‐transporter ER‐ANT1

Leroch *et al*. (2008) reported the discovery of an ER ATP transporter in the plant *Arabidopsis thaliana*. Phylogenetic analysis of the *A. thaliana* genome revealed that the candidate ER adenine nucleotide transporter ER‐ANT1 belongs to the family of mitochondrial carriers (MCF) forming a monophyletic group with mitochondrial ATP/ADP carriers (AACs) (Picault *et al*., 2004) (locus tag At5g17400). Accordingly, ER‐ANT1 features a high sequence similarity of about 60% with AAC1, AAC2, and AAC3, albeit lacking the putative N‐terminal transit peptide required for mitochondrial targeting (Murcha, Millar & Whelan, 2005; Leroch *et al*., 2008). Heterologous expression of ER‐ANT1 in *Escherichia coli*, which involved integration of the protein in the cytoplasmic membrane, was utilized to examine its ability to transport ATP (Leroch *et al*., 2008). The expression of ER‐ANT1 allowed the uptake of radiolabelled ATP and ADP by *E. coli* cells with a high specificity similar to the mitochondrial isoform AAC1 from *A. thaliana*. However, the transport activity of ER‐ANT1 could neither be inhibited by a number of known AAC1 inhibitors nor by inhibitors of plastidic or peroxisomal adenine nucleotide transport but was inhibited by N‐ethylmaleimide, which does not affect the mitochondrial transporter. Further experiments revealed that ER‐ANT1 also mediates the efflux of ATP and ADP, which led to a model of transport involving the exchange of the two nucleotides. In addition to the transport studies in *E. coli*, the *A. thaliana* plant itself was used to demonstrate the localization of ER‐ANT1 in the ER *via* cell fractionation and immunoblotting as well as electron microscopy following immunogold labelling. The expression levels of ER‐ANT1 were tested using the β‐glucuronidase (GUS) reporter system and the results showed elevated promotor activities in tissues with highly active ER metabolism, such as pollen, root tips and seeds. Leroch *et al*. (2008) also examined the physiological role of the ATP transporter by studying ER‐ANT1‐knockout plants. Here the knockout caused a severe growth defect and impaired root and seed development. Moreover, the expression of ER‐resident chaperones was analysed. Intriguingly, the absence of ER‐ANT1 accompanied decreased levels of the luminal binding proteins BiP1, BiP2, and BiP3 as well as calreticulin1 and calreticulin2; mRNA levels of other ATP‐dependent ER proteins were also reduced. These data emphasize the importance of ATP transport into the ER and should encourage the search for a possible mammalian counterpart, which perhaps may be one of several uncharacterized AAC‐like proteins.

### (4) Characterization of ATP dynamics within the ER lumen

Independent of the mode of ATP transport into the ER, several attempts have been made to characterize this process in a variety of circumstances. Certain observations may help in the design of new strategies for the elucidation of the mechanisms of ER ATP import. For example, ER‐targeted genetically encoded ATP sensors now allow the monitoring of ATP dynamics in live cells (Vishnu *et al*., 2014) (see also Section III.7 and Fig. 2B). This approach has revealed that ATP levels within the ER are related to Ca^2+^ levels in that a reduction of Ca^2+^ in the ER causes a significant elevation of ATP levels. Further application of such sensors will certainly reveal other, similar connections.

### (5) A general perspective on ER transport

A broader view of ER transport might contribute to a better understanding of the ER membrane's barrier function and how to overcome it. The import of nucleotide sugars, ATP and nucleotide sulfate into the ER and Golgi was reviewed by Hirschberg *et al*. (1998). A more recent review by Csala *et al*. (2007) summarizes the transport of compounds such as glucose, nucleotide sugars and oligopeptides such as glutathione across the ER membrane. As in the case of ATP, the identification of transporters for other substrates has proved difficult, and it has often only been possible to provide a functional description of transport activities. Most strategies have involved measuring transport into reconstituted proteoliposomes containing ER proteins (see also Section III.7 and Fig. 2A). Among the known transporters are those of the solute carrier (SLC37) family of sugar phosphate/phosphate exchangers (Chou & Mansfield, 2014). This family includes, among others, the SLC37A4 glucose‐6‐phosphate transporter which was found *via* sequence homology with a bacterial transporter for phosphate esters (Gerin *et al*., 1997). The search for transporters can be extended using bioinformatics. Another solute carrier family, SLC35, contains nucleotide sugar transporters resident in the ER and Golgi (Ishida & Kawakita, 2003). Nucleotide sugars are required for the glycosylation of proteins in the ER and Golgi where they are transported by an antiport mechanism in exchange for the corresponding nucleotide monophosphate, e.g. UMP for UDP‐glucose. The overall importance of SLC transporters becomes evident when considering that around 400 human transporter genes have been classified in 52 SLC families (Hediger *et al*., 2003). It is highly likely that an ER ATP transporter will be identified among the SLC proteins with unknown function. Indeed, as described above (Section III.3) the *A. thaliana* ER ATP transporter shows homology with members of the mitochondrial carrier family (AAC), which is denoted SLC25 in the human system. Alternatively, a specific ATP transport protein might not be necessary, since ER‐derived vesicles are permeable to various compounds (Le Gall, Neuhof & Rapoport, 2004). A reasonable explanation for this permeability may be found in the translocon channel (Sec61 complex) which enables the co‐ and post‐translational transport of proteins into the ER (Osborne, Rapoport & van den Berg, 2005; Gogala *et al*., 2014; Dudek *et al*., 2015; Pfeffer *et al*., 2015) but may also allow the rather non‐specific passage of other molecules (Csala *et al*., 2007). Intriguingly, a recent study in yeast, shows that glutathione can enter the ER *via* facilitated diffusion through Sec61, along a concentration gradient (Ponsero *et al*., 2017). To return to ER ATP transport, it seems plausible then that ATP could diffuse through the translocon channel during protein translocation. Furthermore, the coupling of protein and ATP import would make sense, since protein folding requires an ATP supply. Since ATP concentrations are significantly lower in the ER compared to the cytosol and mitochondria (Depaoli *et al*., 2018), a concentration gradient could be the driving force for ATP import.

### (6) ATP transport through anion channels

Since ATP is required in all compartments of the cell, ATP transport mechanisms are also needed. The mitochondrial ATP/ADP‐translocase is described in Section III.3 and a likely vesicular ATP transporter in Section III.2. Regarding the transport of ATP into the Golgi there is some evidence that ATP import can be mediated by Golgi anion transporters, which are mainly involved with the transport of Cl^−^ into the lumen (Thompson *et al*., 2006). Similarly, anion channels of the cardiac sarcoplasmic reticulum (SR) were shown to conduct ATP and other adenine nucleotides (Kawano *et al*., 1999). Nevertheless, the permeability or conduction of ATP through anion channels does not necessarily imply that ATP can be transported in this way in live cells.

### (7) Methods for the investigation of ER ATP dynamics

Studies involving the measurement of ATP transport into reconstituted proteoliposomes have utilized an approach involving radioactively labelled ATP and size‐exclusion chromatography (Fig. 2A) (Mayinger & Meyer, 1993; Guillén & Hirschberg, 1995). First, proteoliposomes are incubated with radiolabelled ATP. The transport reaction is then stopped by loading the mixture onto a size‐exclusion column (Dovex, Sephadex). ATP dissolved in the incubation medium is incorporated into the matrix, while proteoliposomes containing imported ATP are eluted in the void volume. Thus free ATP is separated from ATP found inside the proteoliposomes. The respective ATP concentrations can be measured *via* high‐performance liquid chromatography (HPLC) or liquid scintillation counting and compared to determine the transport velocity.

ATP transport will also be reflected in the change in ATP levels within the ER compared to other cellular compartments and the cytosol, and might thus be indirectly observed when measuring ATP concentrations in living cells. Of course, ATP levels will be affected not only by transport but also by ATP‐consuming and ATP‐producing processes. To monitor ATP dynamics, genetically encoded ATP sensors containing fluorescent proteins have been developed. A major advance in this field was the development of Förster/fluorescence resonance energy transfer (FRET)‐based indicators for ATP (‘ATeams’) consisting of a cyan and a yellow fluorescent protein linked *via* the ϵ‐subunit of the F_o_F_1_‐ATP synthase from *Bacillus subtilis* (Fig. 2B) (Imamura *et al*., 2009). The binding of ATP to the ϵ‐subunit causes a conformational change within the protein, resulting in increased FRET. An ER‐targeted sensor derived from an ATeam protein was constructed by Vishnu *et al*. (2014). For ER targeting, the signal sequence of the ER chaperone calreticulin was fused to its N‐terminus and the ER retention signal composed of the amino acid sequence – lysine (K), aspartic acid (D), glutamic acid (E), and leucine (L) – (KDEL) to its C‐terminus (Pelham, 1990; Clairmont *et al*., 1992). Another ATP probe was developed that used only a single fluorescent protein, circularly permuted enhanced green fluorescent protein (cpEGFP), inserted between two α‐helices of the bacterial F_o_F_1_‐ATP synthase ϵ‐subunit (Yaginuma *et al*., 2014). This ratiometric indicator with the descriptive name ‘quantitative evaluator of cellular energy’ (Queen) undergoes quantifiable changes in its excitation spectrum in response to ATP binding. In an analogous manner to the ATeam sensor, the ER targeting of Queen should be possible. A further fluorescent reporter was found to be suitable for monitoring ATP/ADP ratios (Berg, Hung & Yellen, 2009; Tantama *et al*., 2013).

Intracellular ATP content can also be measured using another phenomenon based upon photon emission: chemiluminescence. The most prominent example is firefly luciferase which uses the energy of ATP to catalyse the light‐producing reaction of luciferin with O_2_ to produce oxyluciferin (Fig. 2C) (DeLuca & McElroy, 1974). The intensity of emitted light is proportional to ATP concentration if the other substrates are present in excess. Purified luciferase can be added together with luciferin to permeabilized cells or isolated mitochondria to measure ATP levels (Manfredi *et al*., 2002). However, the heterologous expression of recombinant luciferase also allows a live‐cell approach (Ainscow & Rutter, 2002; Bell *et al*., 2007), and intracellular ATP levels can be described in more detail by targeting luciferase to different compartments (Dorner & Kaufman, 1994; Jouaville *et al*., 1999; Kennedy *et al*., 1999). Applications of the luciferase system were reviewed by Fan & Wood (2007) and Patergnani *et al*. (2014).

Measurements of the total content of ATP, ADP and AMP within the cell may also help investigators to draw conclusions about the origin of changes in ATP levels. A convenient method is HPLC, which allows the simultaneous quantification of all nucleotide species. The use of HPLC in this way is reviewed by Manfredi *et al*. (2002) who also outline correct sample handling, which is required for successful analysis to avoid the degradation of ATP by ATPases in the sample.

### (8) The source of ER ATP

ATP is generated during glycolysis and oxidative phosphorylation. The contribution of different metabolic pathways to ATP production may vary in different cell types and under different conditions including ER stress, cell cycle, aging or carcinogenesis. In the case of cancer cells, which have an altered energy metabolism, it is tempting to speculate that there may also be abnormalities in the ER ATP supply. In general, cancer cells produce most of their ATP *via* high rates of aerobic glycolysis [the ‘Warburg effect’; reviewed by Liberti & Locasale, 2016], while normal cells have lower rates of glycolysis and preferentially use oxidative phosphorylation for ATP generation. Accordingly, the source of ER ATP and perhaps also the import mechanism might vary depending on the metabolic state of the cell. It seems likely that, in cells with high rates of mitochondrial respiration, ATP generated within the mitochondria is directly transferred into the ER, possibly *via* MAM contact sites, while glycolytic ATP might rather enter from the cytosol.

## IV. CONCLUSIONS

**Figure 3:**
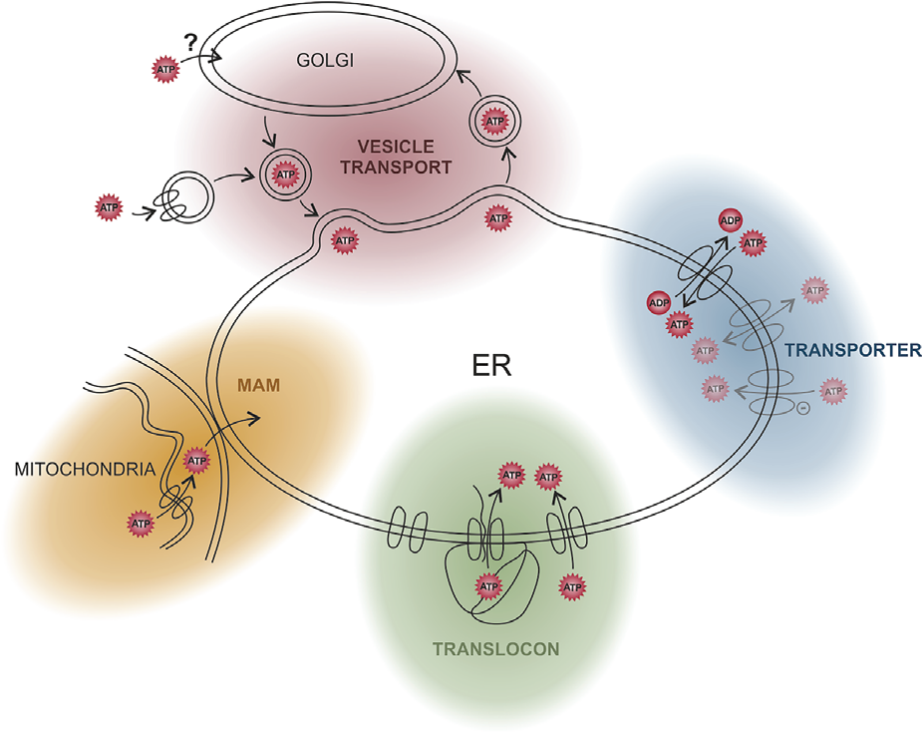
Possible ATP transport routes into the endoplasmic reticulum (ER). It is not known how ATP is transported into the ER, but several possible import pathways are shown. Experiments with rat liver microsomes provided some evidence for a specific ATP transporter and an ATP/ADP antiport mechanism. ATP may also be transported non‐specifically through anion channels. The ER membrane may also be ‘leaky’ for ATP, allowing its passive transport driven by a concentration gradient. One such source of leakiness may be translocon channels, which are widely distributed across the membrane of the rough ER; ATP could enter the ER concomitantly with the inserted peptide or by a translocon‐associated mechanism. Since ATP is mainly produced in mitochondria it seems reasonable that it is directly transported from there into the ER *via* membrane contact sites (mitochondria‐associated ER membranes; MAMs). Finally, the dynamics of the membrane structures evoked by membrane trafficking *via* vesicles might also afford the opportunity for ATP uptake.

(1) The adequate supply of the ER with ATP is important for its functions in protein synthesis and turnover, functions that underlie the role of the ER in cell growth. ATP is produced during glycolysis in the cytosol and oxidative phosphorylation in mitochondria; the ER itself is not capable of ATP generation. Thus ATP utilized by the ER must be transported there by a still‐unknown mechanism.

(2) Possible ER ATP import routes are summarized in Fig. 3. The idea that a specific ATP transporter is involved is plausible, especially given that the mitochondrial ATP/ADP‐translocase enables the transfer of ATP from its place of origin within the mitochondria to other cell compartments. However, equating the ER with mitochondria is probably not prudent, since these organelles are quite different in their architecture, function, and origin (Archibald, 2015). However, the ER is related to the Golgi: both are part of the endomembrane system (Dacks & Field, 2007). The fact that a specific ATP transporter has not been identified in either of these two compartments may indicate that no such transporter is present.

(3) The dynamics of the ER or Golgi membrane structures during membrane trafficking events might also afford the opportunity for ATP uptake.

(4) Membrane contact sites such as mitochondria‐associated ER membranes (MAMs) could serve as a platform for ATP transfer, possibly even directly from mitochondria into the ER.

(5) ATP hydrolysis likely follows ATP import rapidly and thus an ATP concentration gradient could provide a driving force for passive diffusion through transient membrane breaks (see point 3 above), or proteinaceous channels. Indeed, ATP concentrations within the ER are considerably lower than in the cytosol and mitochondria. Passive transport could be allowed by the leakiness of the ER, caused by the great number of translocon channels spanning the membrane. However, even if the ER is not leaky for ATP, the translocon might still be associated with ATP import in a translocon‐coupled mechanism which would allow the coordination of ATP supply with the energy‐consuming processes of protein translocation and folding.

(6) Irrespective of which of these mechanisms (and perhaps several are involved) are actually responsible for ATP import into the ER, this review aims to stimulate discussion that has the potential to yield a wealth of new insights into molecular cell biology. We look forward to future developments in this field.
